# FAM46B inhibits cell proliferation and cell cycle progression in prostate cancer through ubiquitination of β-catenin

**DOI:** 10.1038/s12276-018-0184-0

**Published:** 2018-12-10

**Authors:** Tao Liang, Xuxiao Ye, Yuanyuan Liu, Xinkai Qiu, Zuowei Li, Binqiang Tian, Dongliang Yan

**Affiliations:** 1 Department of Urology, Shanghai Sixth People’s Hospital East Affiliated to Shanghai University of Medicine & Health Sciences, Shanghai, 201306 China; 2 Department of Urology, Affiliated Hospital of Binzhou Medical University, Binzhou, 256603 China

## Abstract

FAM46B is a member of the family with sequence similarity 46. Little is known about the expression and functional role(s) of FAM46B in prostate cancer (PC). In this study, the expression of FAM46B expression in The Cancer Genome Atlas, GSE55945, and an independent hospital database was measured by bioinformatics and real-time PCR analysis. After PC cells were transfected with siRNA or a recombinant vector in the absence or presence of a β-catenin signaling inhibitor (XAV-939), the expression levels of FAM46B, C-myc, Cyclin D1, and β-catenin were measured by western blot and real-time PCR. Cell cycle progression and cell proliferation were measured by flow cytometry and the CCK-8 assay. The effects of FAM46B on tumor growth and protein expression in nude mice with PC tumor xenografts were also measured. Our results showed that FAM46B was downregulated but that β-catenin was upregulated in patients with PC. FAM46B silencing promoted cell proliferation and cell cycle progression in PC, which were abrogated by XAV-939. Moreover, FAM46B overexpression inhibited PC cell cycle progression and cell proliferation in vitro and tumor growth in vivo. FAM46B silencing promoted β-catenin protein expression through the inhibition of β-catenin ubiquitination. Our data clearly show that FAM46B inhibits cell proliferation and cell cycle progression in PC through ubiquitination of β-catenin.

## Prostate cancer: getting earlier diagnosis

A little-studied protein may help in early diagnosis and treatment of prostate cancer (PC), one of the most common cancers in men. Because early-stage PC causes few symptoms, many patients are not diagnosed until later stages, when treatment options are limited. New methods for early diagnosis and treatment are actively sought. Proteins in the FAM46 family are known to be involved in many types of cancer. Dongliang Yan at Shanghai Sixth People’s Hospital East and co-workers investigated what role one protein in this family, FAM46B, might play in PC. Analysis of tumor samples showed that FAM46B levels were much lower in PC than in healthy tissues. These changes were linked to another tumor-associated protein, β-catenin. In further tests in mice, artificially increasing FAM46B levels decreased tumor size. These results could improve treatments for PC.

## Introduction

Prostate cancer (PC) is one of the most common malignant tumors of the male urogenital system. The incidence of PC is the second highest of all malignant tumors in males worldwide and therefore poses a serious threat to men’s health^1^. In some European and American countries, the incidence of PC varies according to race and lifestyle; in some cases, the incidence of PC is even higher than that of lung cancer and is thus the most common cause of cancer-related death^2^. Although the incidence and detection rates of PC in China are lower than those in western countries, the incidence has increased in recent years^3^. As those with early-stage PC generally have no obvious symptoms, the majority of patients are often in the late clinical stage when distant bone metastasis has already occurred;^4^ this results in the inability to perform radical surgery, which is associated with a poor prognosis and contributes to the higher mortality rate in China compared with other countries. Therefore, it is of great significance to further explore the mechanism of PC and to find new methods by which PC can be diagnosed and treated early.

Family with sequence similarity 46 member B (FAM46B) belongs to a family of four genes (FAM46A, FAM46B, FAM46C, and FAM46D) and has been found to be involved in tumorigenesis. It was found that FAM46A expression is significantly decreased in patients with PC^5^ and may be an independent predictive risk factor for non-small cell lung cancer and breast cancer^6,7^. As a tumor suppressor, FAM46C functions in the regulation of cell proliferation, apoptosis, and metastasis of hepatocellular carcinoma,^8,9^ and multiple myeloma^10,11^. FAM46D, despite its unknown function, is expressed in prostate carcinoma PC-3 cells and in other cancers, including gastric, breast, brain, lung, and gynecological cancers^12^. FAM46B expression was found to be lower in metastatic melanoma cells (United States Patent US 7615349 B2) and serves as a potential marker for multiple myeloma^13^ and refractory lupus nephritis^14^. However, the expression and pathological function of members of the FAM46 family, especially FAM46B, in PC are not fully understood.

The classical Wnt/β-catenin signaling pathway has been a hotspot in the field of molecular biology in recent years. Abnormal activation of the Wnt/β-catenin signaling pathway has been implicated in the occurrence of a variety of cancers, such as colorectal^15^, breast^16^, liver,^17^ and lung cancers^18^. β-catenin is the most important transduction factor in the Wnt signaling pathway, which participates in intercellular adhesion and the regulation of cell growth, differentiation, and apoptosis^19^. β-catenin binds to the nuclear transcription factor TCF to form a complex that regulates downstream C-myc and Cyclin D1 expression and induces malignant transformation in cells^20,21^ to fulfill the effect of Wnt signaling. Moreover, the suppression of β-catenin ubiquitination has been found to be associated with the progression of hepatocellular carcinoma^22^ as well as colorectal^23^, gastric,^24^ and prostate cancers^25^. These results indicate that β-catenin ubiquitination and degradation may play an important role in the progression of PC.

In the present study, we sought to determine whether cell proliferation, cell cycle progression, and apoptosis in PC were regulated by FAM46B in vitro and in vivo. In addition, the β-catenin signaling pathway response to FAM46B-induced PC tumorigenesis was also investigated. We found that FAM46B was downregulated in PC and inhibited PC cell cycle progression and cell proliferation through ubiquitination of β-catenin. Our study provides further proof-of-principle that FAM46B targeting could be an effective clinical approach to prevent PC progression.

## Materials and methods

### Tissue samples

In all, 100 tumor tissues as well as 30 adjacent noncancerous tissues were obtained from PC patients who were recruited from March 2013 to October 2016 at Shanghai Sixth People’s Hospital East Affiliated with Shanghai University of Medicine & Health Sciences. All of the patients provided signed informed consent. The medical ethics committee of the hospital approved the present retrieval method of the cancer specimens.

### Bioinformatics

The gene expression data were obtained from the NCBI Gene Expression Omnibus (GEO, Access id: GSE GSE55945) and The Cancer Genome Atlas (TCGA, https://tcga-data.nci.nih.gov/tcga/).

### Cell culture and transfection

PC-3, LNCaP, and DU145 human PC cell lines, and RWPE-1 and P69 prostate epithelial cell lines were obtained from American Type Culture Collection (Manassas, VA, USA). PC-3 cells were cultured in F-12K medium (GIBCO BRL, Gaithersburg, MD, USA). DU145 and RWPE-1 cells were cultured in Rosewell Park Memorial Institute 1640 (Invitrogen Life Technologies, Carlsbad, CA, USA). LNCaP and P69 cells were cultured with Dulbecco’s Modified Eagle’s Medium (Invitrogen Life Technologies) supplemented with 10 mm glucose (Hyclone, Logan, UT, USA). The cells were cultured in medium containing 10% fetal bovine serum (GIBCO) and 1% antibiotic (mixtures of streptomycin and penicillin, Solarbio, Beijing, China) and were maintained in a humidified incubator at 37 °C and 5% CO_2_.

The RNAi (RNA interference) sequences targeting position 358–376 (siFAM46B-1; TCTTCTGAGCGAGCCGATT), position 762−780 (siFAM46B-2; GCAAGAACGTGGAGCTCAA) or 1370–1388 (siFAM46B-3; CCAGCCACTGTCAATTACT) of the human FAM46B gene were transfected into P69 cells using Lipofectamine 2000 (Invitrogen Life Technologies) according to the manufacturer’s protocol. The FAM46B lentiviral vector for ectopic expression was constructed by integrating the coding sequence (CDS) of FAM46B into the pLVX-Puro lentivirus vector. The primers used to synthesize the CDS were as follows: forward: 5′-GCGAATTCATGATGCCGTCGGAGAGC-3′ (*Eco*RI); reverse: 5′-CGGGATCCTCAGTTACAAGGCAGCCAGGTG-3′ (*Bam*HI). The underscore indicates the restriction enzyme cutting site. In brief, 293 T cells were grown in a 6-well plate and were transfected with pLVX-Puro-FAM46B at 37 °C for 5 h using Lipofectamine reagent (Invitrogen Life Technologies). Then, 48 h after transfection, recombinant lentiviral vectors were collected and used for the infection of LNCaP and PC-3 cells. Cells transfected with scramble siRNA (siNC) or blank pLVX-Puro (vector) were used as negative controls.

### Cell proliferation analysis

To assess cell proliferation, a Cell Counting Kit (CCK)-8 (Dojindo Laboratories, Kumamoto, Japan) assay was performed. In brief, P69, PC-3, and LNCaP cells at a density of 3 × 10^3^ cells/well were cultured according to a standard procedure in a 96-well plate and were maintained in a 5% CO_2_ incubator at 37 °C overnight. After 0, 24, 48, and 72 h, CCK-8 solution (10 μl per well) was added to P69, LNCaP, and PC-3 cells transfected with siFAM46B-1, siFAM46B-2, or pLVX-Puro-FAM46B with or without XAV-939 (20 μm; Selleck, Houston, TX, USA) treatment. The cells were then maintained in an incubator with CO_2_ for 1 h at 37 °C, after which the absorbance readings were obtained at 450 nm.

### Cell cycle analysis

P69, PC-3, and LNCaP cells at a density of 5 × 10^5^ cells/well were cultured in six-well plates and maintained at 37 °C for 1 day. For the cell cycle assay, P69, LNCaP, and PC-3 cells transfected with siFAM46B-1, siFAM46B-2, or pLVX-Puro-FAM46B with or without XAV-939 (20 μm) treatment for 48 h were centrifuged at 1000 × *g* for 5 min. The cells were then fixed in 700 μl pre-cooled absolute ethyl alcohol, incubated with 1 mg/ml of RNase A (100 μl; Solarbio) in dark for half an hour and stained with 50 μg/ml propidium iodide (PI, 400 μl; Sigma-Aldrich, St. Louis, MO, USA) for 10 min.

### Quantitative real-time PCR

Total RNA from PC tissues and cell lines was extracted using TRIzol (Invitrogen Life Technologies). Complementary DNA (cDNA) was synthesized using a PrimeScript reagent kit (TaKaRa Biomedical Technology (Beijing) Co., Ltd., Beijing, China) in accordance with the manufacturer’s protocols. Quantitative real-time PCR using SYBR Green (Takara Biomedical Technology (Beijing) Co., Ltd) was performed using the GeneAmp PCR System 2700 (Applied Biosystems Life Technologies, Foster City, CA, USA). The primers used in the present study are as follows: FAM46B-F, 5′-CTGGCTGCCTTGTAACTG-3′ and FAM46B-R, 5′-TCGGGAAAGTCTGGTCTG-3′; CTNNB1-F, 5′-CCTCCAGGTGACAGCAATCAG-3′ and CTNNB1-R, 5′-GCCCTCTCAGCAACTCTACAG-3′ GAPDH-F, 5′-AATCCCATCACCATCTTC-3′ and GAPDH-R, 5′-AGGCTGTTGTCATACTTC-3′. The internal control for mRNA is given as the ratio to GAPDH. The relative quantification was calculated using the 2^−ΔΔCt^ cycle threshold method.

### Western blotting

Total protein was extracted with radio immunoprecipitation assay lysis buffer (Solarbio) and was then centrifuged at 12,000 × *g* for 20 min at 4 °C. Next, 10% sodium dodecyl sulfate polyacrylamide gel electrophoresis was performed to isolate the proteins. After the protein was transferred to nitrocellulose membranes, the membranes were blocked with 5% non-fat milk and then incubated with antibodies against FAM46B (Proteintech, Chicago, IL, USA), Cyclin D1 (Abcam, Cambridge, MA, USA), C-myc (Abcam), β-catenin (Abcam), and GAPDH (Cell Signaling Technology, Inc., Danvers, MA, USA) overnight at 4 °C. The membranes were washed and subsequently incubated with horseradish peroxidase-labeled Goat Anti-Mouse, Donkey Anti-Goat or Goat Anti-Rabbit IgG (Beyotime Institute of Biotechnology, Haimen, China) for 2 h at 25 °C. Signals were detected using an enhanced chemiluminescence western blotting substrate (Pierce; Thermo Fisher Scientific.).

### Co-immunoprecipitation (Co-IP) and ubiquitination

In brief, cold phosphate-buffered saline) was used to wash the cells three times, after which the cells were scraped into lysis buffer containing complete protease inhibitors and were centrifuged at 14,000 × *g* for 20 min at 4 °C. The supernatants were incubated with anti-β-catenin (Abcam; 1:1000) or normal IgG (Abcam; 1:1000) antibody, and the immunocomplexes were then associated with protein A-sepharose. Anti-β-catenin (Abcam; 1:1000) and anti-ubiquitin (Abcam; 1:1000) antibodies were used for the western blot analysis.

### Animal experiments

LNCaP cells (5 × 10^5^) transfected with pLVX-Puro-FAM46B or blank pLVX-Puro were subcutaneously injected into each of six nude mice (4–5-week-old BALB/c mice). Tumor volume (mm^3^) was calculated according to the following standard formula: (the longest diameter) × 0.5 × (the shortest diameter)^2^. After 33 days, the mice were sacrificed, and the tumor tissues were excised and weighed. Animal experiments were performed according to the legal requirements and were approved by the Shanghai Sixth People’s Hospital East Affiliated with Shanghai University of Medicine & Health Sciences Institutional Ethical Committee.

### Statistical analysis

All the results are presented as the mean ± standard deviation, and each test was repeated at least three times. All statistical analyses were performed with GraphPad Prism software using one-way analysis of variance followed by Dunnett’s post hoc test. When *P* < 0.05, the difference between groups was statistically significant.

## Results

### The expression of FAM46B is downregulated in PC tissues and cell lines

**Fig. 1:**
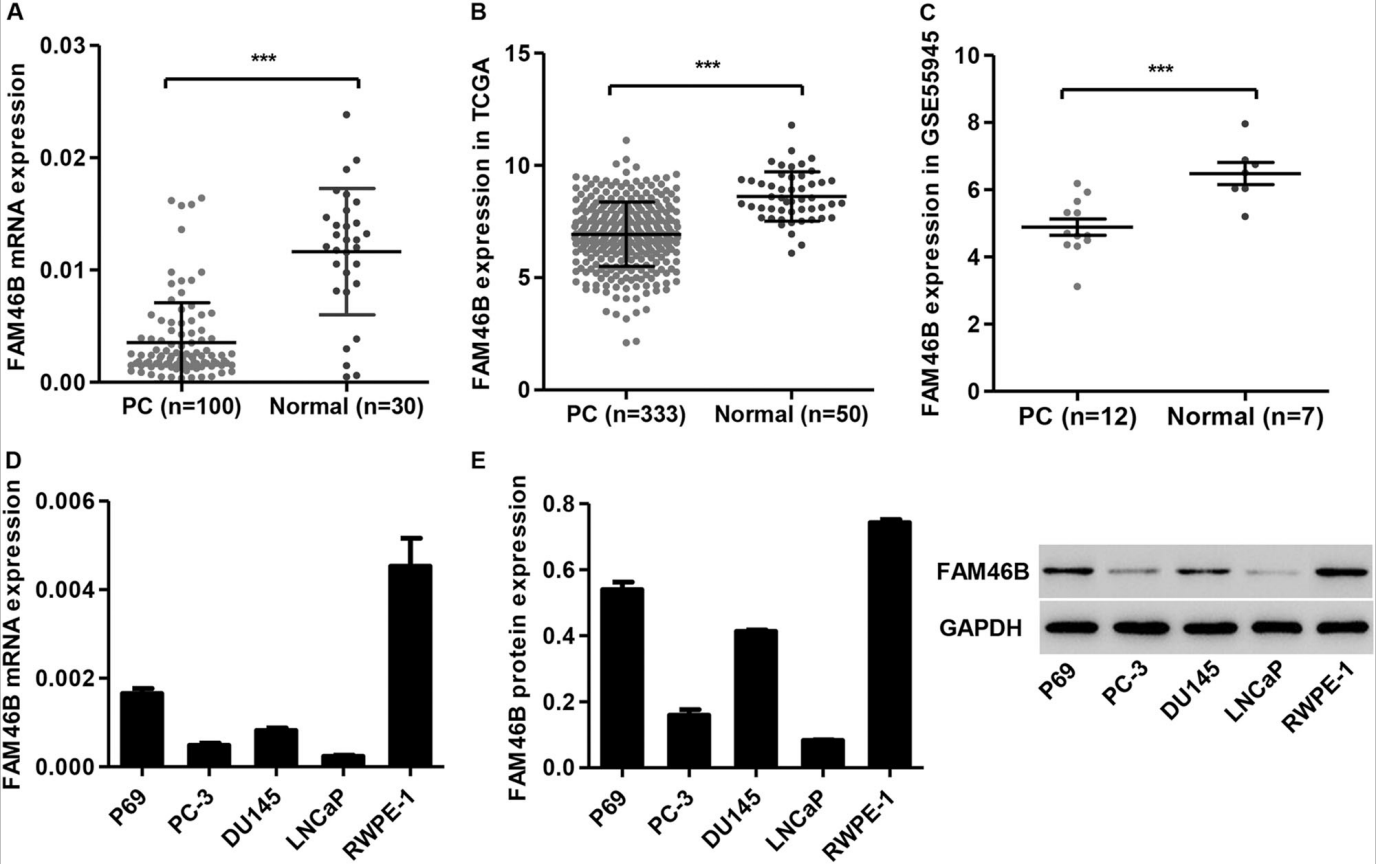
FAM46B expression in PC tissues and cell lines The mRNA expression of FAM46B in PC tissues and corresponding normal prostate tissues collected from an independent hospital **a**, TCGA **b**, and the GSE55945 **c** data set was measured by real-time PCR and bioinformatics analysis. The FAM46B mRNA and protein expression in PC cell lines and prostate epithelial cell lines was measured by real-time PCR **d** and western blotting **e**

Analysis of tissues collected from our independent hospital demonstrated that the mRNA expression of FAM46B in PC tissues (*n* = 100) was downregulated compared with the corresponding normal prostate tissues (*n* = 30) (Fig. 1a). Similarly, the expression of FAM46B in PC tissues obtained from TCGA and the GSE55945 data set was also downregulated compared with normal prostate tissues (Fig. 1b, c). Moreover, the FAM46B expression levels in PC cell lines were also measured by western blotting and real-time PCR. As shown in Fig. 1d, e, the FAM46B mRNA expression in PC cell lines, including PC-3, DU145, and LNCaP cells, was downregulated compared with prostate epithelial cell lines, including P69 and RWPE-1; the lowest expression was detected in LNCaP and PC-3 cells. These results suggest that FAM46B may be related to PC progression.

### Construction of FAM46B RNAi and overexpressing cell lines

**Fig. 2:**
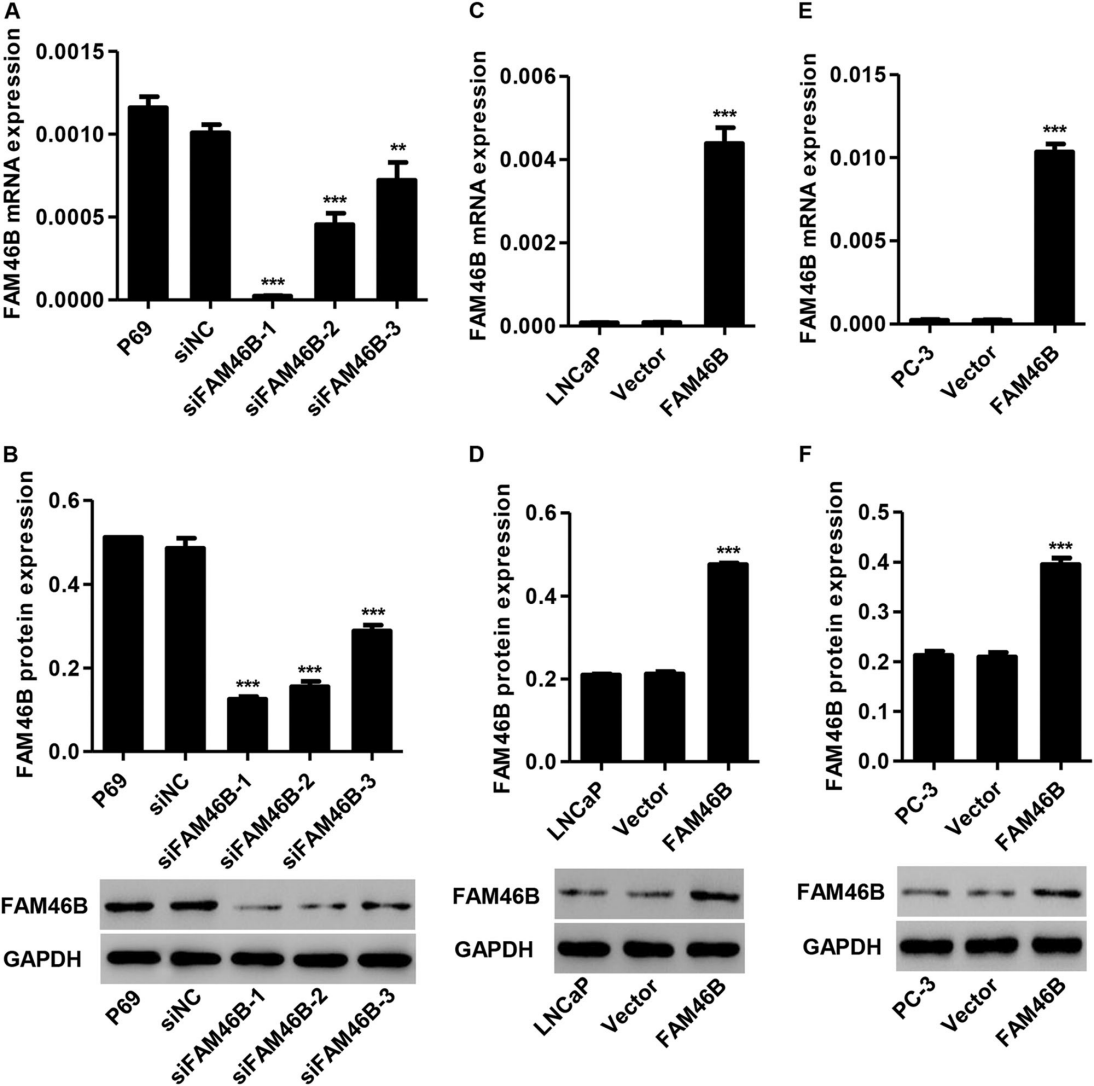
Construction of FAM46B RNAi and overexpressing cell lines Real-time PCR and western blotting were performed to examine the mRNA and protein expression of FAM46B in P69 cells **a**, **b** transfected with siFAM46B-1, siFAM46B-2, or siFAM46B-3, and in LNCaP **c**, **d** and PC-3 cells **e**, **f** transfected with pLVX-Puro-FAM46B. **P < 0.01, ***P < 0.001 compared with siNC or blank vector

P69 cells were transfected with three siRNAs targeting human FAM46B or a siNC. FAM46B silencing significantly reduced the mRNA and protein expression of FAM46B. siFAM46B-1, siFAM46B-2, and siFAM46B-3 transfection inhibited FAM46B protein expression by 74.2%, 68.0, and 40.5%, respectively, compared with siNC (Fig. 2a, b). Therefore, siFAM46B-1 and siFAM46B-2 were used in the following in vitro experiments. In addition, LNCaP and PC-3 cells were transfected with the recombinant pLVX-Puro-FAM46B vector or blank pLVX-Puro. As shown in Fig. 2c, d, FAM46B overexpression in LNCaP cells significantly increased the mRNA and protein expression of FAM46B by 50.5-fold and 1.25-fold, respectively, compared with the blank vector. FAM46B overexpression in PC-3 cells significantly increased the mRNA and protein expression of FAM46B by 44.2-fold and 0.88-fold, respectively, compared with the blank vector (Fig. 2e, f).

### FAM46B silencing promotes P69 cell proliferation and cell cycle progression

**Fig. 3:**
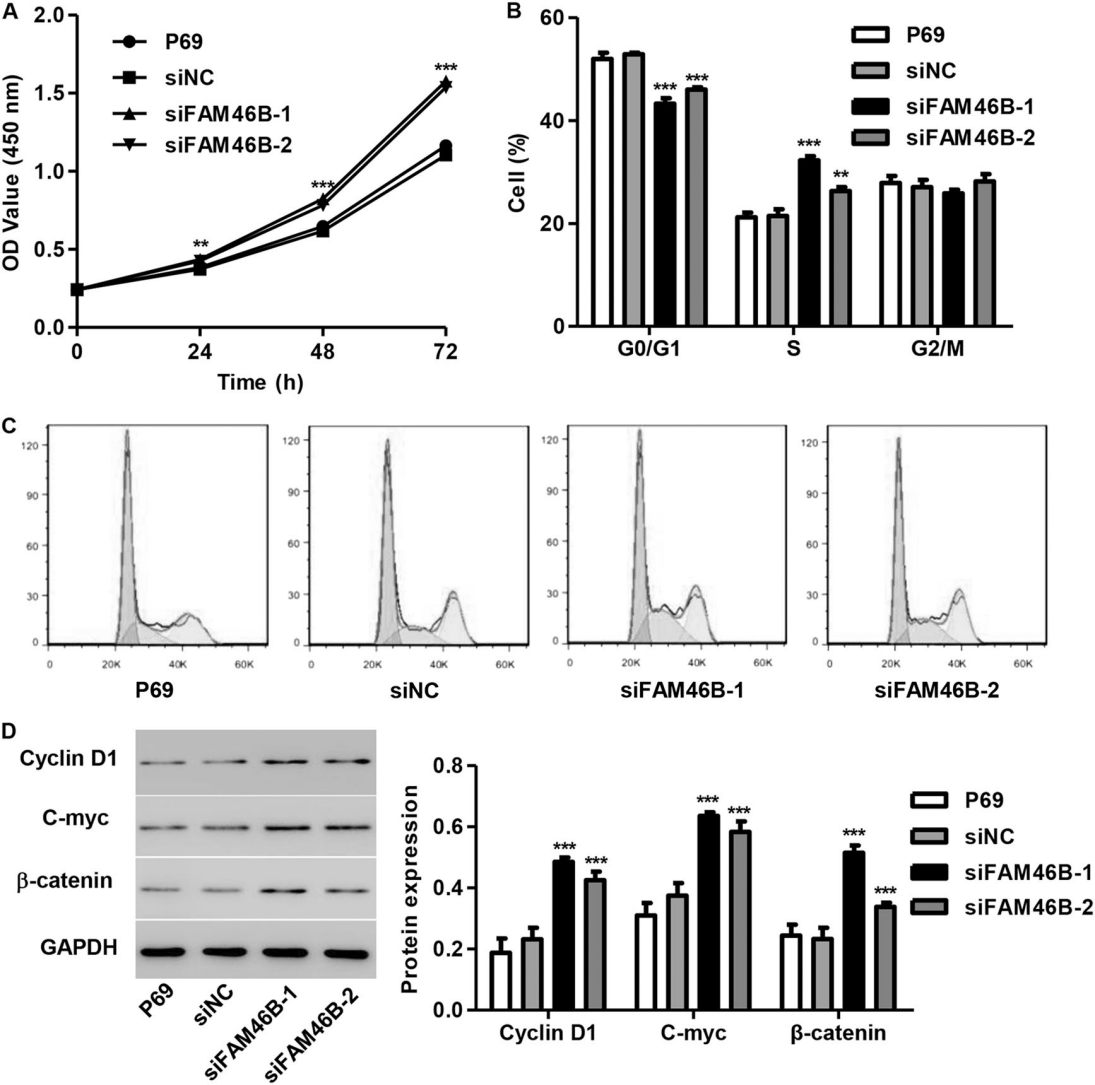
FAM46B silencing promoted cell proliferation and cell cycle progression in P69 cells After P69 cells were transfected with siFAM46B-1 and siFAM46B-2, cell proliferation, cell cycle progression and the expression of C-myc, Cyclin D1, and β-catenin were measured by CCK-8 assay **a**, flow cytometry **b**, **c**, and western blotting **d**, respectively. **P < 0.01, ***P < 0.001 compared with siNC

After P69 cells were transfected with siFAM46B-1 or siFAM46B-2, cell cycle progression and cell proliferation were measured by flow cytometry and CCK-8 assay. We found that siFAM46B-1 and siFAM46B-2 significantly promoted the proliferation of P69 cells by 16.6 and 13.9% at 24 h, by 33.3% and 26.5% at 48 h, and by 42.8 and 39.2% at 72 h, respectively, compared with siNC (Fig. 3a). Moreover, siFAM46B-1 and siFAM46B-2 significantly decreased the number of cells in G0/G1 phase by 18.1% and 11.5%, respectively, and increased the number of cells in S phase by 50.5% and 22.7%, respectively, compared with siNC (Fig. 3b, c). In addition, the transfection of P69 cells with siFAM46B-1 and siFAM46B-2 significantly increased the protein expression of C-myc, Cyclin D1, and β-catenin compared with siNC (Fig. 3d).

### FAM46B overexpression inhibits PC cell proliferation and blocks cell cycle progression

**Fig. 4:**
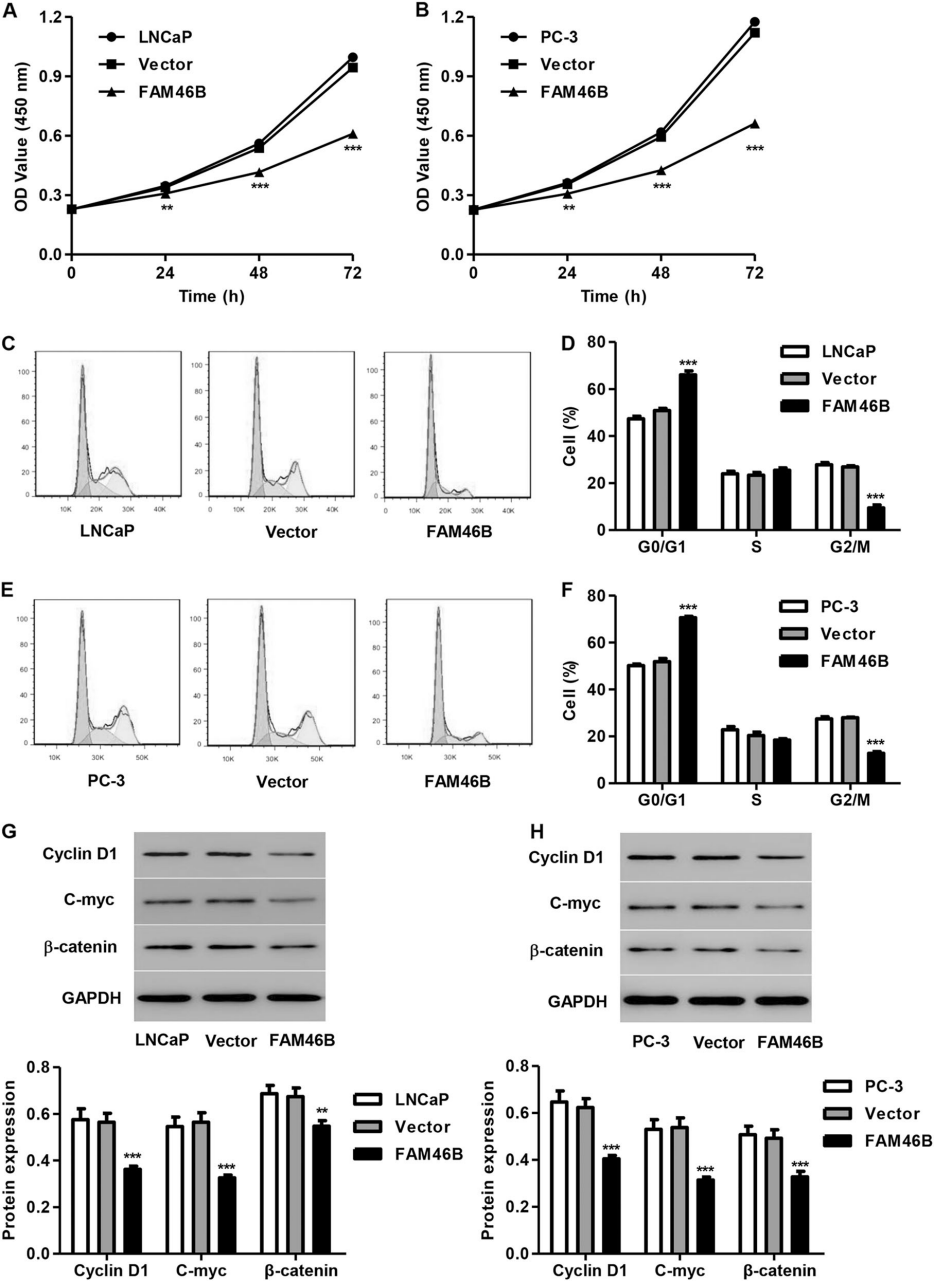
FAM46B overexpression inhibited cell proliferation and cell cycle progression in LNCaP and PC-3 cells After LNCaP and PC-3 cells were transfected with pLVX-Puro-FAM46B, cell proliferation, cell cycle progression and the expression of C-myc, Cyclin D1, and β-catenin were measured by CCK-8 assay **a**, **b**, flow cytometry **c**–**f**, and western blotting **g**, **h**, respectively. **P < 0.01, ***P < 0.001 compared with blank vector

After LNCaP and PC-3 cells were transfected with pLVX-Puro-FAM46B, cell proliferation and cell cycle progression were also measured. We found that transfection of cells with pLVX-Puro-FAM46B significantly inhibited the proliferation of LNCaP and PC-3 cells by 9.1 and 13.5% at 24 h, by 22.7% and 28.3% at 48 h, and by 35.4 and 40.9% at 72 h, respectively, compared with the blank vector (Fig. 4a, b). Moreover, the transfection of LNCaP and PC-3 cells with pLVX-Puro-FAM46B significantly decreased the proportion of cells in G2/M phase by 64.9 and 54.3%, respectively, and increased the proportion of cells in G0/G1 phase by 29.7 and 36.1%, respectively, compared with the blank vector (Fig. 4c–f). In addition, the transfection of PC-3 and LNCaP cells with pLVX-Puro-FAM46B significantly decreased the expression of Cyclin D1, C-myc, and β-catenin proteins compared with the blank vector (Fig. 4g, h). These results indicate that FAM46B regulates PC cell cycle progression and cell proliferation through Cyclin D1, C-myc, and β-catenin signaling.

### FAM46B overexpression inhibits PC tumor growth in vivo

**Fig. 5:**
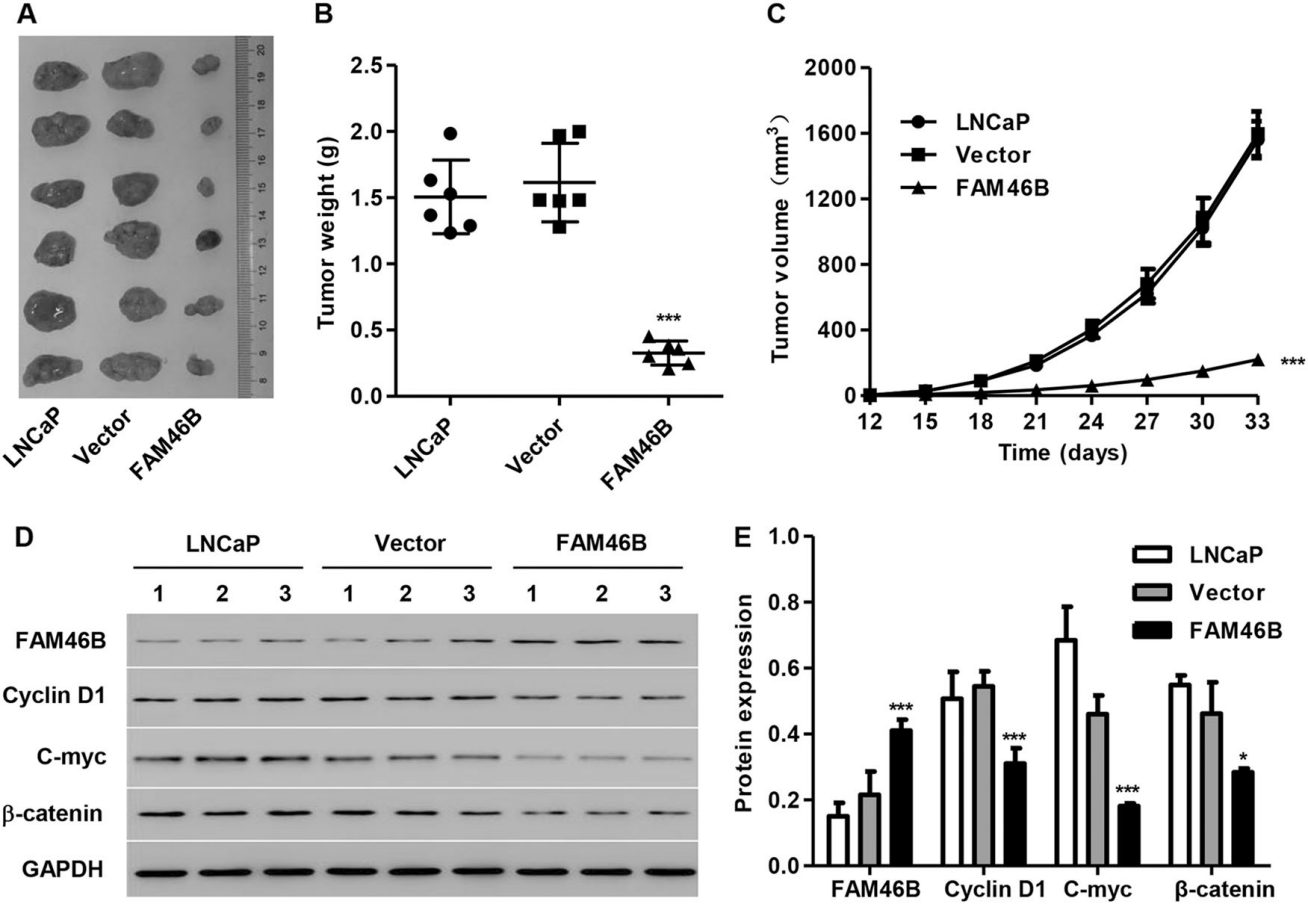
FAM46B overexpression inhibited PC tumor growth in vivo After LNCaP cells were transfected with pLVX-Puro-FAM46B virus, they were injected into nude mice to establish a xenograft model; after 33 days, the tumor weight **a**, **b** and volume **c** were evaluated, and the protein expression of FAM46B, C-myc, Cyclin D1, and β-catenin were measured in xenograft tumors **d**, **e** by western blotting. *P < 0.05, ***P < 0.001 compared with blank vector

To examine the effect of FAM46B on PC tumorigenesis in vivo, LNCaP cells transfected with pLVX-Puro-FAM46B or blank vector were injected into nude mice. We found that mice injected with pLVX-Puro-FAM46B had decreased tumor weights and smaller tumor volumes (Fig. 5a–c). The protein expression levels of C-myc, Cyclin D1, and β-catenin were significantly decreased in xenograft tumors from mice injected with pLVX-Puro-FAM46B compared with those from mice injected with blank vector (Fig. 5d, e).

### XAV-939 treatment inhibits FAM46B silencing-induced PC cell proliferation and cell cycle progression

**Fig. 6:**
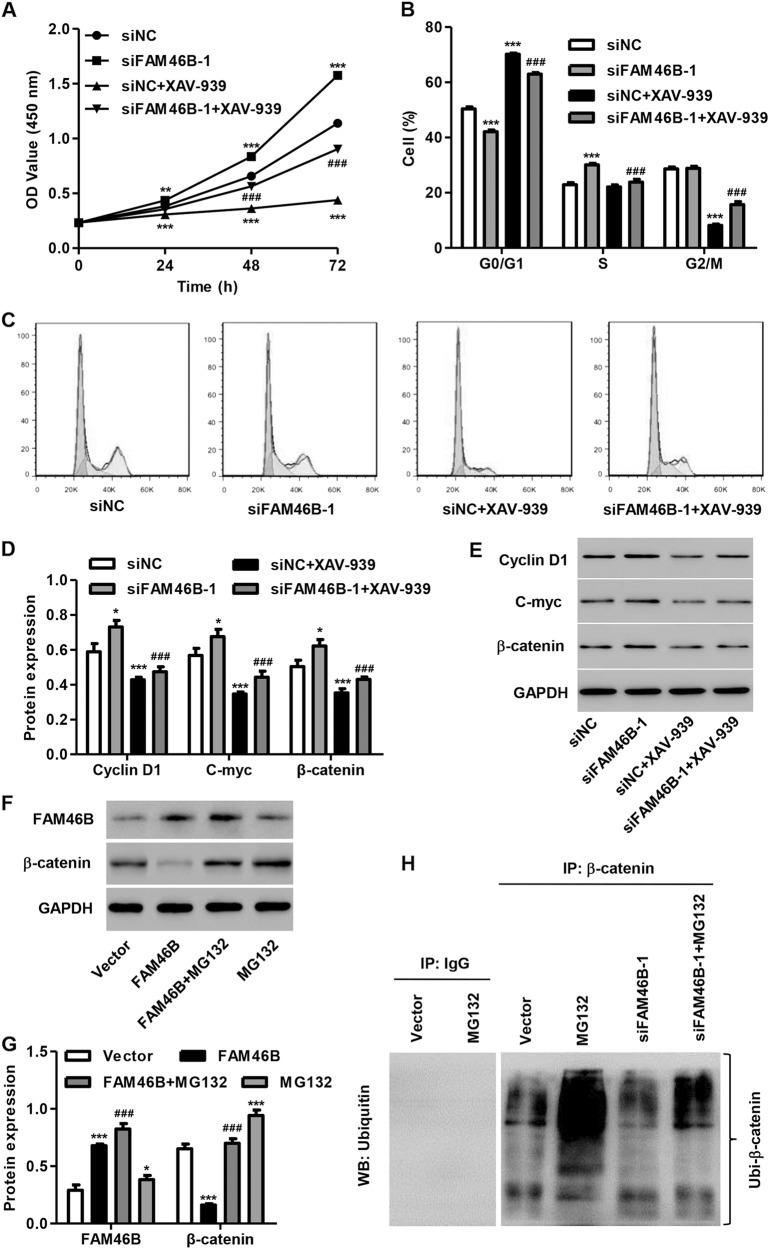
XAV-939 treatment inhibited FAM46B silencing-induced PC cell proliferation and cell cycle process After P69 cells were transfected with siFAM46B-1 with or without XAV-939 treatment (20 μm), cell proliferation and cell cycle progression were measured by CCK-8 assay **a** and flow cytometry **b**, **c**, and the expression of C-myc, Cyclin D1, and β-catenin was measured by western blotting **d**, **e**. PC-3 cells transfected with pLVX-Puro-FAM46B were treated with MG132 (50 μm) for 4 h, and the expression of FAM46B and β-catenin proteins was measured by western blotting **f**, **g**. β-catenin was immunoprecipitated and immunoblotted in P69 cells transfected with siFAM46B-1 with or without MG132 (50 μm) treatment for 4 h **h**. *P < 0.05, **P < 0.01, ***P < 0.001 compared with siNC or blank vector. ^###^*P* < 0.001 compared with siFAM46B-1 or FAM56B

To investigate the involvement of β-catenin signaling in FAM46B-induced cell cycle progression and cell proliferation in PC, P69 cells transfected with siFAM46B-1 were treated with the β-catenin signaling inhibitor XAV-939 (20 μm), after which cell proliferation and cell cycle progression were measured. We demonstrated that XAV-939 treatment significantly inhibited the siFAM46B-1 transfection-induced increase in cell proliferation, the proportion of cells in S phase and the decrease in the proportion of cells in G0/G1 phase (Fig. 6a–c). Moreover, XAV-939 treatment significantly inhibited the siFAM46B-1 transfection-induced increase in the expression of Cyclin D1, C-myc, and β-catenin proteins (Fig. 6d, e). However, FAM46B silencing or overexpression did not affect the mRNA expression of β-catenin (data not shown), which suggests that FAM46B may regulate the post-transcriptional level of β-catenin. As shown in Fig. 6f, g, FAM46B overexpression in PC-3 cells significantly decreased β-catenin protein expression, which was reversed by treatment with MG132, a proteasome inhibitor. This indicates that FAM46B may regulate β-catenin protein expression in a proteasome-dependent manner. Moreover, Co-IP and ubiquitination analysis were performed and showed that FAM46B silencing significantly inhibited β-catenin ubiquitination, which was reversed by MG132 treatment (Fig. 6h); this suggests that FAM46B ubiquitinates β-catenin.

### Correlation analysis between FAM46B and β-catenin in PC tissues

**Fig. 7:**
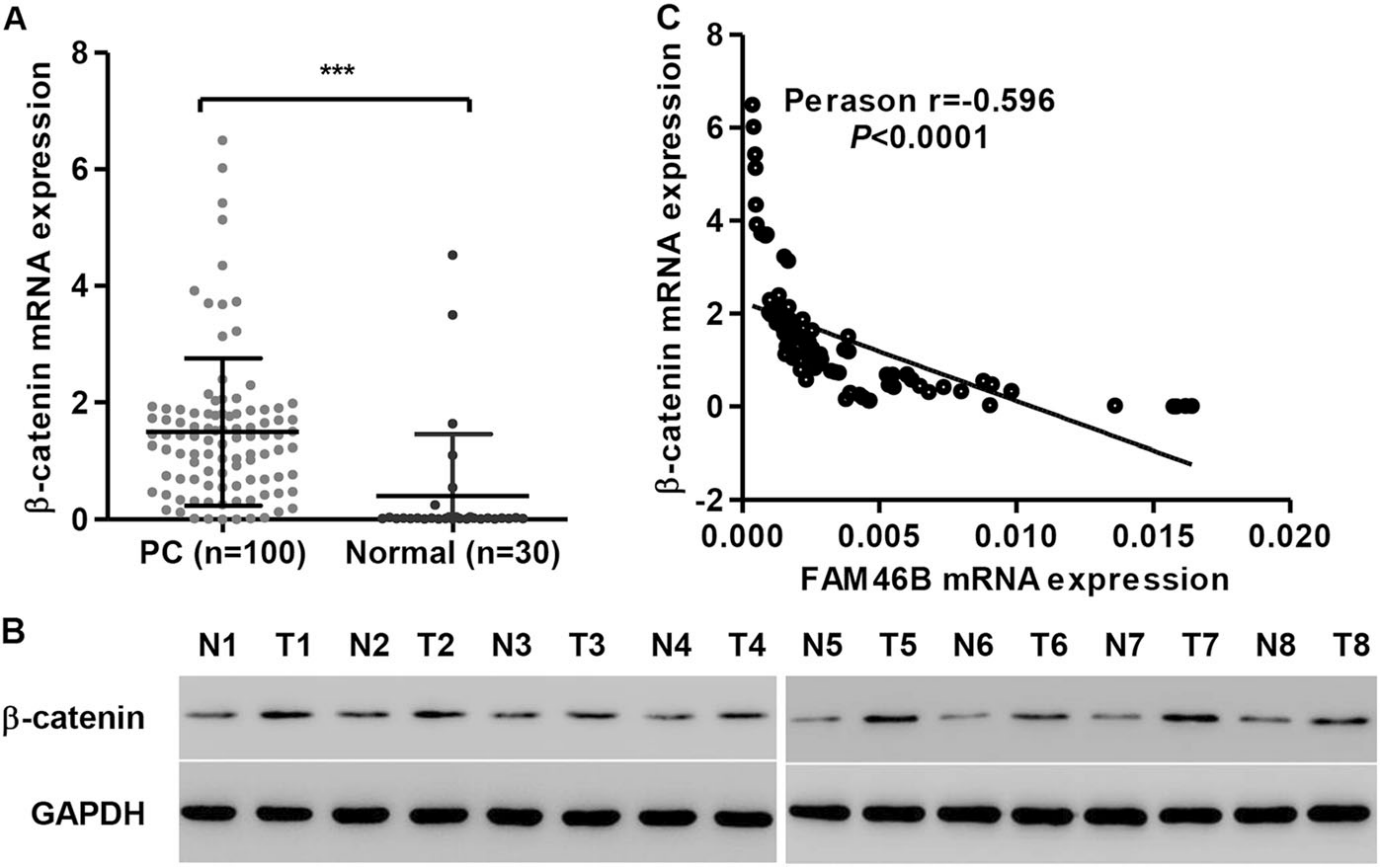
FAM46B was negatively correlated with β-catenin in PC tissues The mRNA and protein expression of β-catenin in PC tissues and corresponding normal prostate tissues was measured by real-time PCR **a** and western blotting**b**. Linear regression showed that FAM46B mRNA expression was negatively correlated with the mRNA expression of β-catenin**c**. N1-8: normal prostate tissues; T1-8: PC tissues

β-catenin protein and mRNA expression was measured by western blotting and real-time PCR, respectively. As shown in Fig. 7a, b, β-catenin expression in PC tissues was upregulated compared with normal prostate tissues. Moreover, linear regression showed that FAM46B mRNA expression was negatively correlated with β-catenin mRNA expression in PC tissues (Fig. 7c). These data further supported the findings in PC cell lines.

## Discussion

In the present study, we found that FAM46B expression was downregulated, whereas β-catenin expression was upregulated in PC patients from an independent hospital, TCGA and the GSE55945 data set. FAM46B overexpression inhibited PC cell cycle progression and cell proliferation in vitro and PC tumor growth in vivo through the inhibition of β-catenin signaling via ubiquitination.

FAM46B is currently known as a member of the FAM protein family. The expression and biological function of FAM46B in PC tumorigenesis are largely unknown. However, roles in the regulation of apoptosis, the cell cycle, and cell proliferation have been suggested for other FAMs. FAM46C and FAM43B induced apoptosis and cell proliferation inhibition in multiple myeloma and hepatocellular carcinoma cell lines^8,10,26^, and FAM176A induced cell cycle arrest in non-small cell lung cancer cells^27^. Given that all FAM members exhibit a high degree of sequence homology, they might share functional patterns. In the present study, FAM46B was found to be downregulated in PC tissues obtained from our hospital, which was consistent with the analysis based on data from the TCGA, the GSE55945 data set, and PC tissue microarrays. A previous study demonstrated decreased FAM46B expression in metastatic melanoma cells, which suggests the involvement of FAM46B in cancer development and progression^13^.

Cyclin D1 is an oncogene that regulates cell cycle function through combination with CDK4 or CDK6 in G1 phase and through binding to retinoblastoma protein, which results in its phosphorylation at serine and tyrosine residues. This in turn leads to the release of the transcription factor E2F, after which cyclin D1 promotes the smooth transition of cells from G1 phase to S phase, cell growth, and differentiation^28–30^. The abnormal expression of Cyclin D1 disturbs the cell cycle and induces tumorigenesis. As a transcription factor, C-myc can activate the expression of cell cycle-related genes including Cyclin E, Cyclin D2, and Cyclin D1, but can repress p27 expression^31,32^, so that p27 can be isolated from Cyclin E; cells are then able to transition from G1 phase to S phase^33^. In the present study, we found that FAM46B overexpression induced cell cycle arrest in G0/G1 phase and inhibited cell proliferation and the expression of Cyclin D1 and C-myc both in vitro and in vivo, whereas FAM46B silencing demonstrated the inverse effect. These findings suggest that FAM46B regulates PC cell cycle progression and cell proliferation through the modulation of C-myc and Cyclin D1 expression. These data were in agreement with our findings on the roles of Cyclin D1 and C-myc in PC cell proliferation and cell cycle progression^34,35^. However, downregulation of C-myc and Cyclin D1 expression in PC-3 cells was associated with S and G2/M phase arrest rather than G0/G1 phase arrest^36^. This may be explained by the possibility that the induction of cell cycle arrest is not only dependent on the regulation of C-myc and Cyclin D1 but also on other cell cycle regulatory factors.

We also examined the molecular mechanism by which FAM46B inhibits PC tumorigenesis. Wnt/β-catenin is a relatively conserved signaling pathway in the process of biological evolution. This pathway participates in the regulation of pathological and physiological processes in the cell such as proliferation, differentiation, and cell death. Continuous activation of Wnt/β-catenin signaling leads to excessive stem cell renewal or proliferation, which can easily lead to tumor induction^37^. Our results demonstrated that β-catenin expression was upregulated in PC tissues and was negatively correlated with the expression of FAM46B in PC tissues, which was in agreement with our in vitro study. Moreover, the inhibition of β-catenin in PC cells by treatment with XAV-939 significantly inhibited FAM46B knockdown-induced cell proliferation, cell cycle regulation, and the expression of Cyclin D1 and C-myc. These data indicate that β-catenin signaling may be involved in the regulation of PC tumorigenesis by FAM46B. Similarly, the β-catenin/C-myc/Cyclin D1 signaling pathway was also found to be involved in PC and in the tumorigenesis of other cancers^20,21,38^. Furthermore, our Co-IP and ubiquitination analysis confirmed the correlation between FAM46B and β-catenin with respect to FAM46B ubiquitination of β-catenin. A previous study also showed that suppression of β-catenin ubiquitination inhibited PC tumorigenesis^25^. Accordingly, we speculated that FAM46B inhibits PC cell proliferation and cell cycle progression in PC through ubiquitination of β-catenin.

In summary, this study indicates that FAM46B is downregulated in PC tissues. FAM46B overexpression significantly induces the cell cycle arrest and cell proliferation inhibition in PC cells through ubiquitination of β-catenin. Our study may provide insights that advance our understanding of the occurrence and development of PC.
